# Supplementary material for: Mechanosensitive channel engineering: A study on the mixing and matching of YnaI and MscS sensor paddles and pores
Source: Nat Commun. 2025 Aug 23;16:7881. doi: 10.1038/s41467-025-63253-0 (PMC12375004; doi:10.1038/s41467-025-63253-0)
Supplement: Supplementary file 1 — Supplementary Information [file 41467_2025_63253_MOESM1_ESM.pdf]

	YnaI WT	YnaI WT	YnaI WT	YnaI A155V	YnaI A155V	YnaI A155V
condition	I+	II	III	I	II	III
Purification details	Stand. DDM + lipids	LMNG	High DDM	Stand. DDM	LMNG	High DDM
State	closed	closed	Open (45%),	closed	closed	Open (60%)
2D class averages	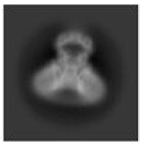	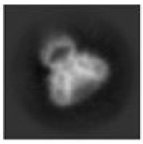	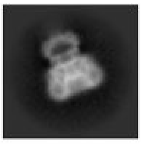	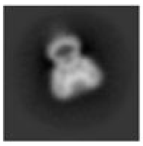	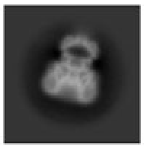	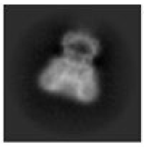
Real space slices from cryoSPARC	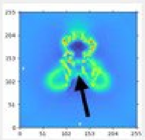	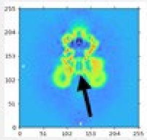	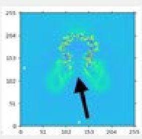	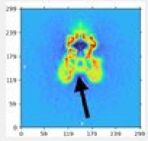	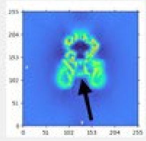	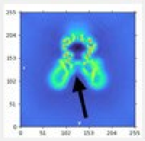

Figure S1: Summary of the different purification approaches.

The table describes the different purification strategies applied to WT-YnaI and YnaI^{A155V} and the observed state of the purified protein after cryo-EM analysis. Furthermore, representative 2D class averages and map projections from the cryoSPARC[1] output are shown. On a 2D class average level, the closed and open conformation are not distinguishable, but the map slice (taken from cryoSPARC) of the open conformation lacks the second density bridge within the periplasmic entrance of the pore (black arrows).

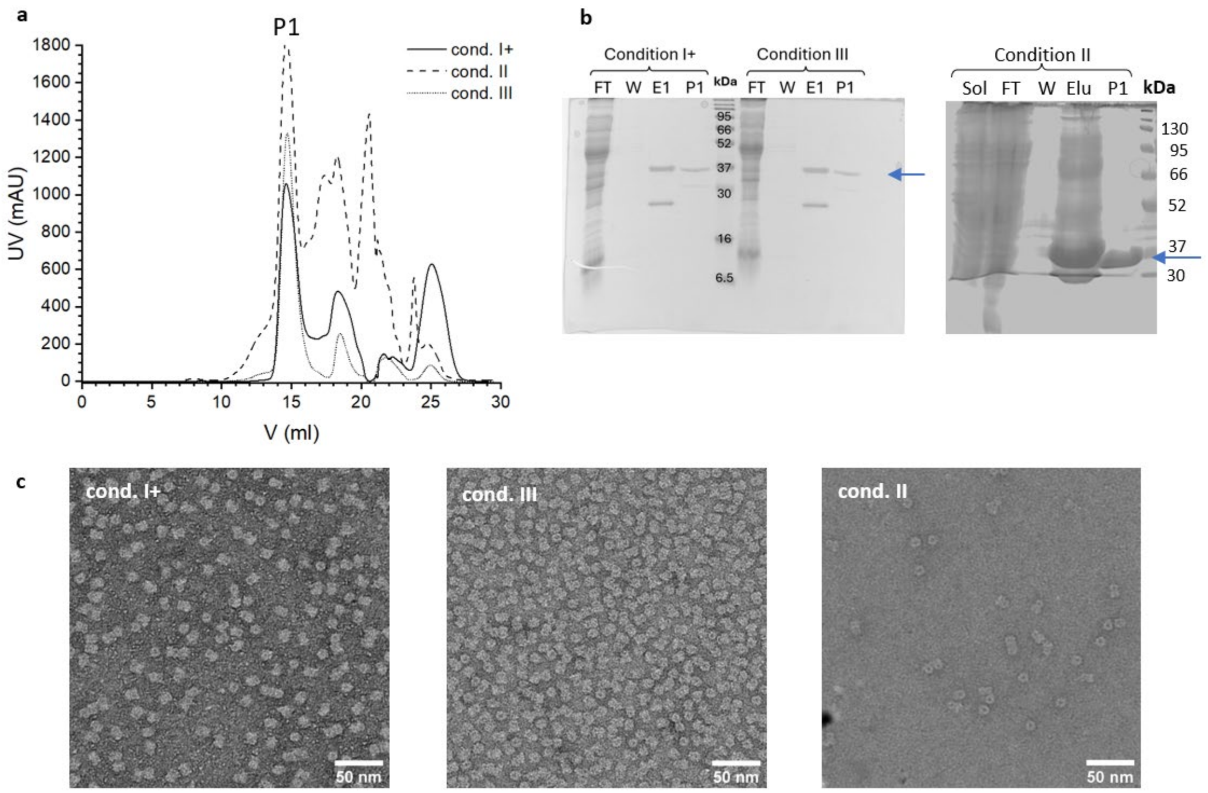

Figure S2: Purifications of WT-YnaI under the different conditions.

Wt-YnaI was purified under different conditions. **a** The size exclusion chromatograms are shown for YnaI purified under condition I+ (standard DDM concentrations, solid line), II (LMNG, dashed line), and III (high DDM concentrations, dotted line). The peak at 15 ml (P1) is the desired heptamer. **b** SDS-PAGEs are shown for all three purification approaches, samples were taken from the supernatant after solubilisation (Sol), IMAC flowthrough (FL), IMAC wash (W), IMAC protein-containing elution fraction (Elu), and HPLC heptamer peak (P1), as well as a Marker (kDa). The signals from YnaI at the height of the 37 kDa marker signal are highlighted with a blue arrow. **c** The P1 fractions were pooled and concentrated for subsequent cryo-EM preparation. The sample was negatively stained with 2% uranyl acetate and imaged for quality assessment. Source data are provided as a source data file.

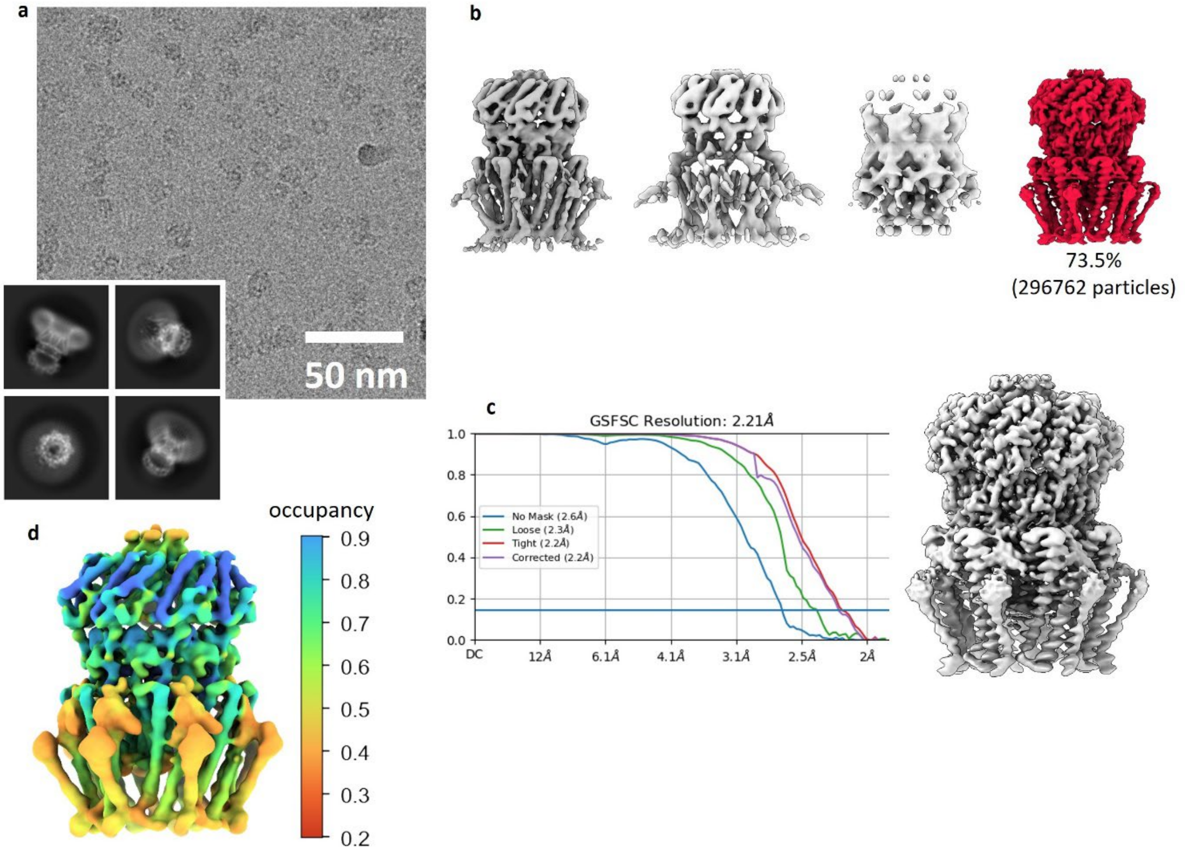

Figure S3: Cryo-EM analysis of closed WT-YnaI purified under condition I+.

a One representative micrograph of the data collection is shown and four selected 2D class averages. The edge of the box corresponds to 24.2 nm. **b** A heterogenous refinement revealed one major class (red) that was further processed. **c** A non-uniform refinement resulted in a map at 2.2 Å resolution. Data processing was done in cryoSPARC.[1] **d** Occupancy estimation of the map was performed with OccuPy[2], showing lower values for the outer cytosolic loop and the helix TM(-2).

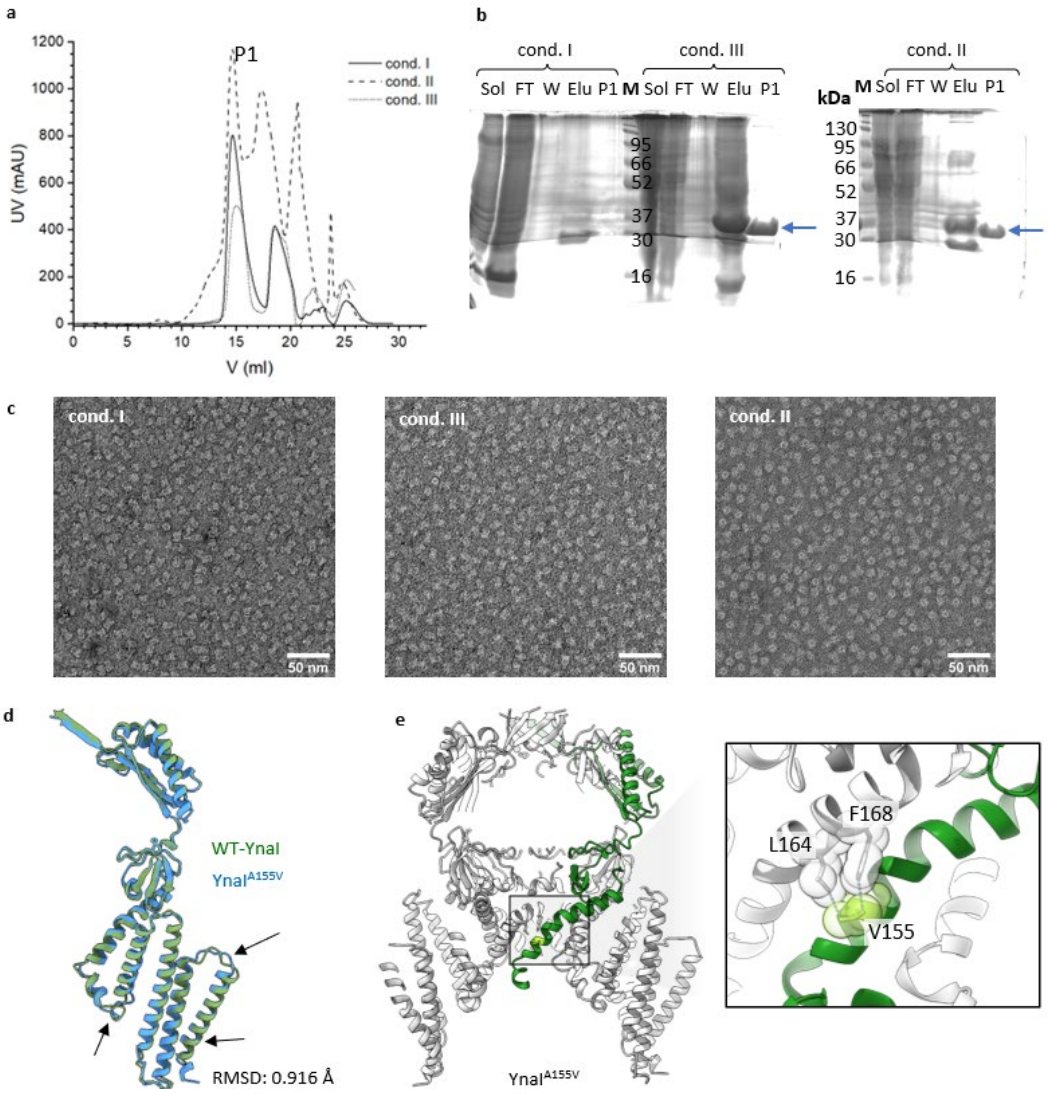

Figure S4: Purifications of YnaI^{A155V} under the different conditions.

YnaI^{A155V} was purified under different conditions. **a** The size exclusion chromatograms are shown for YnaI^{A155V} purified under condition I+ (standard DDM concentrations, solid line), II (LMNG, dashed line), and III (high DDM concentrations, dotted line). The peak at 15 ml (P1) is the desired heptamer. **b** SDS-PAGEs were done to monitor the purification. For all three purification approaches, samples were chosen from the supernatant after solubilisation (Sol), IMAC flowthrough (FL), IMAC wash (W), IMAC protein-containing elution fraction (Elu), and HPLC heptamer peak (P1), as well as a Marker (M). The signals from YnaI at the height of the 37 kDa marker signal are highlighted with a blue arrow. **c** The P1 fractions were pooled and concentrated for subsequent cryo-EM preparation. The sample was negatively stained with 2% uranyl acetate and imaged for quality assessment. **d** The models of one chain of open wt-YnaI (green) and open YnaI^{A155V} (blue) are superposed. The RMS deviation of the two models is 0.916 Å, and the major differences (black arrows) are found in the weak resolved loops and the outermost helix TM(-2). **e** A slice through YnaI^{A155V} is shown with one subunit depicted in green with the side chains of the substitutive Val155 coloured in light green. The black box indicates the enlarged area. It shows the interaction between the Cγ atoms of Val155 with the side chains of Leu164 and Phe168 that is absent in WT-YnaI. For better illustration, transparent van-der-Waals-radii are shown for the mentioned residues. Source data are provided as a source data file.

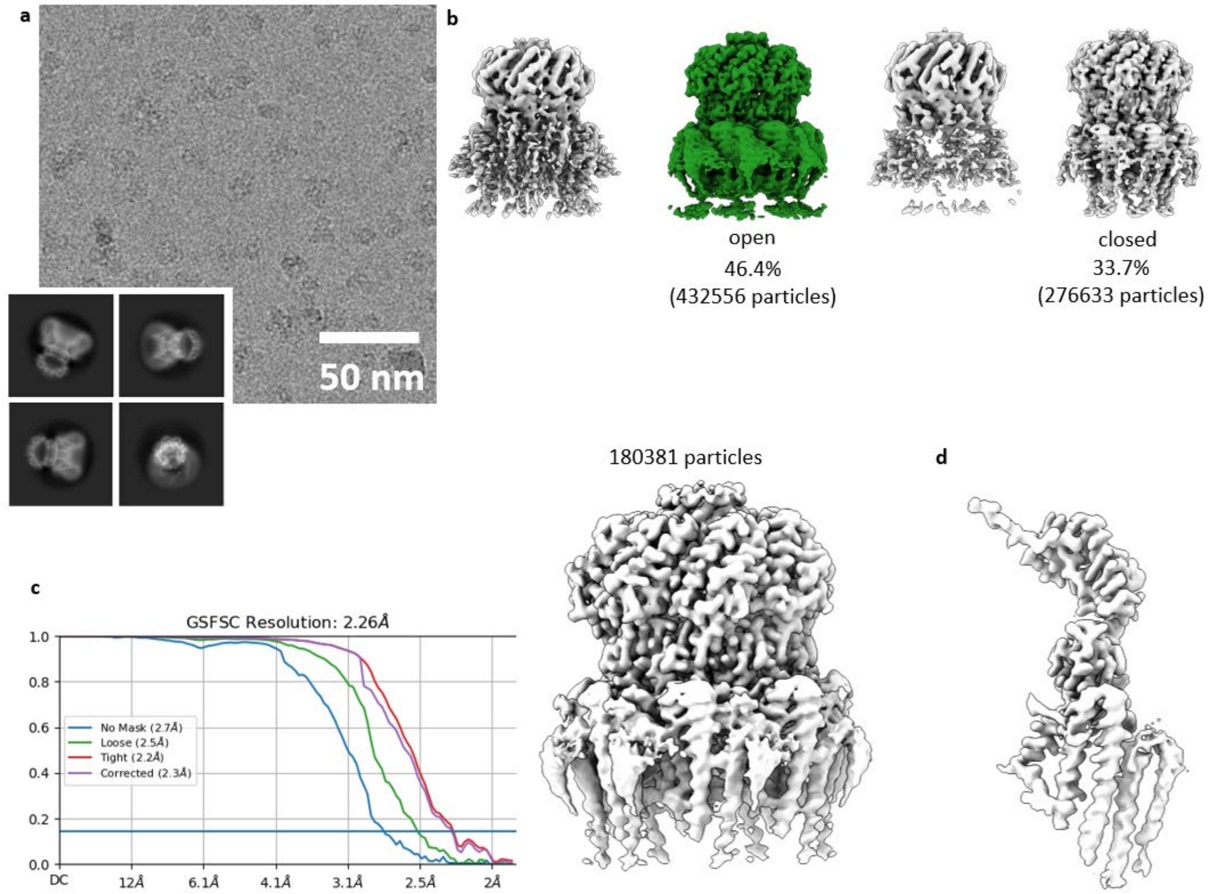

Figure S5: Cryo-EM analysis of open WT-YnaI.

a One representative micrograph of the data collection is shown together with representative 2D class averages. The edge of the box corresponds to 24.2 nm. **b** A heterogenous refinement revealed one open class (green) corresponding to approx. 46 % of all particles. This class was further processed. A closed class was also observed, containing approx. 34 % of all particles. **c** A non-uniform refinement resulted in a map at 2.3 Å resolution. Data processing was done in cryoSPARC.[1] **d** To improve resolution and map quality of helix TM(-2), data was imported into Relion 5[3] and a refinement with C7 symmetry relaxation and blush denoising was conducted. The resulting density of the best resolved subunit is shown.

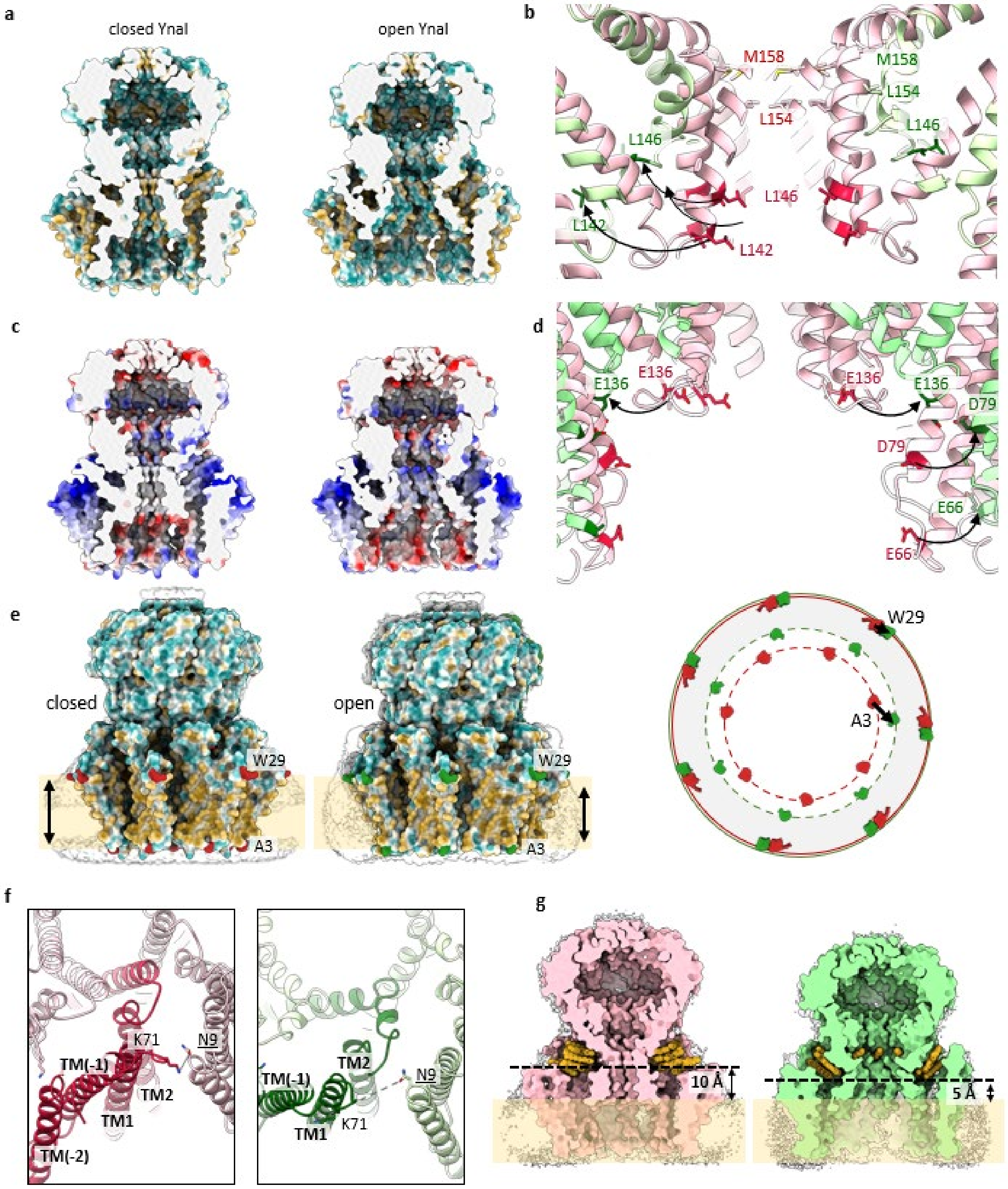

Figure S6: Comparison of the area change of the TM part of closed and open YnaI.

a The surface of the models of closed (left) and open (right) YnaI was coloured by hydrophobicity in ChimeraX.[4,5] The front of the model is clipped for better visualisation of the pore. **b** Apart from the known hydrophobic constriction comprised of Met158 and Leu154, there are also Leu146 and Leu142 (red) in closed YnaI (pink) facing towards the symmetry axis. In the open conformation (light green) these leucines (green) protrude away from the channel axis, because the periplasmic part of the helix TM3a bends outwards. **c** The electrostatic potential of the surface is shown for closed and open YnaI as visualized by ChimeraX.[4,5] The front of the model is clipped for better visualisation of the pore. This highlights the accumulation of negatively charged residues at the periplasmic indentation. **d** The inner surface of the periplasmic indentation is highly electronegative, owing to the residues Glu66, Aps79, and Glu136 (red) which face towards the pore axis ion the closed conformation (pink). As the indentation becomes wider in the open conformation (light green), these residues (green) are spaced more and partly rotate away from

the pore axis. **e** On the left, the surfaces of closed and open YnaI are coloured by their hydrophobicity. The significant residues Trp29, which marks the membrane interface at the cytosolic leaflet, and Ala3, which sits at the periplasmic side, are colored in red (closed YnaI) and green (open YnaI), respectively. In white, the map density is shown at a low threshold, highlighting the position of the detergent micelles. The yellow bars and black arrows depict the approximate membrane position and thickness. On the left, the highlighted residues are viewed from the periplasmic side, showing that the diameter of the channel at the cytosolic side (solid lines; red – closed; green – open) does not change upon opening, but at the periplasmic side (dotted lines). **f** YnaI is viewed from the periplasmic side. One subunit of closed (pink) and open (light green) YnaI is highlighted in a darker colour, and the TM helices are labelled. As only inter-subunit interaction in the paddles, Lys71 and Asn9 interact via H bonds in the closed state. In the open state, the paddles are radially relocated as rigid body. This results in further spacing of the paddles, and the H bond cannot be maintained. **g** The surfaces of closed (pink) and open (green) YnaI schown. The corresponding map densities are shown behind the surface representations, and the membrane plane is indicated by a transparent yellow rectangle. For an estimation of the displacement of the hydrophobic pockets, the position of the pocket lipids closest to the cytosolic leaflet is shown as a black dotted line and the distance is measured. While the pocket lipids in the closed state are approx. 10 Å away from the membrane, the distance is only 5 Å in the open state.

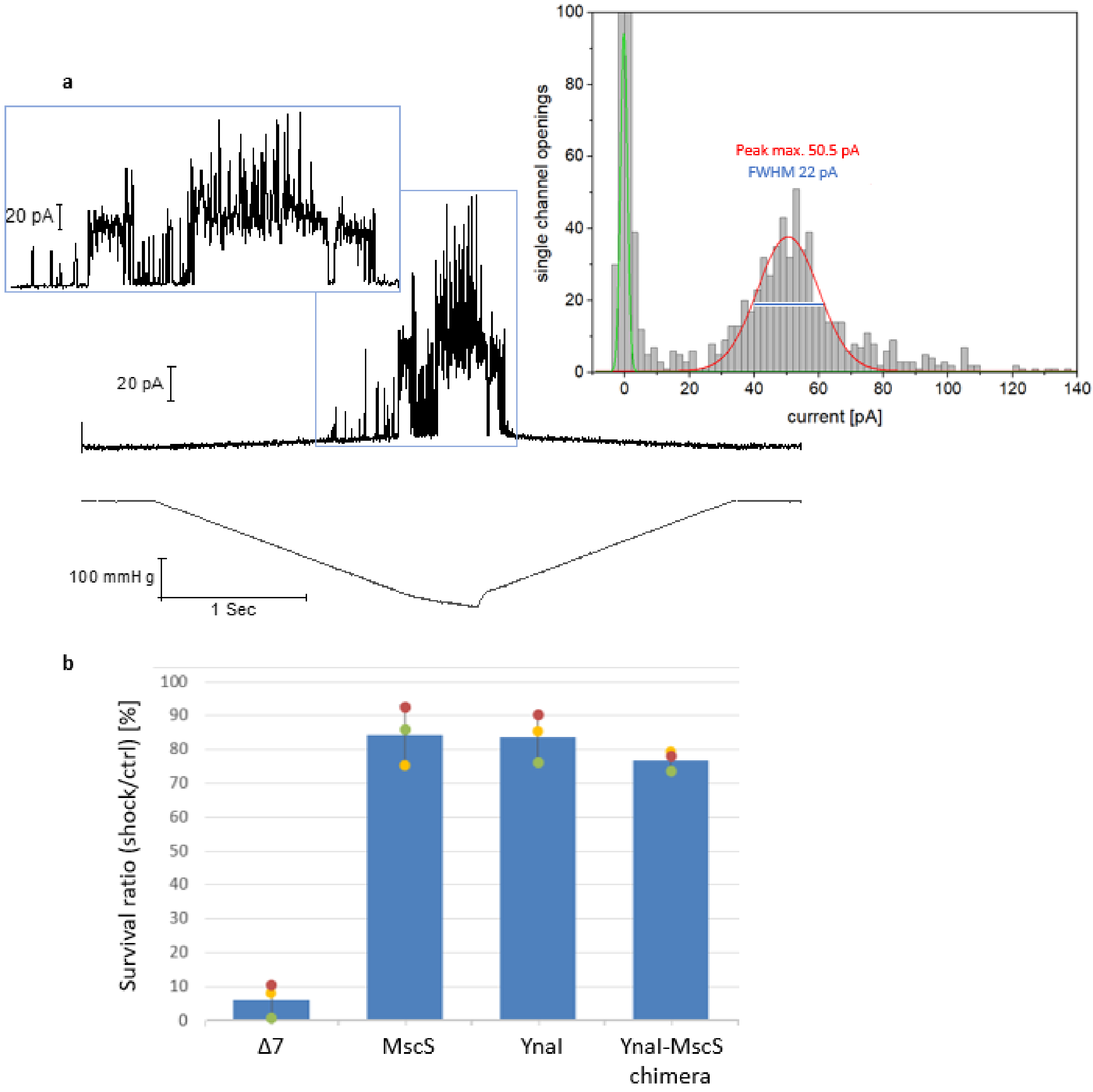

Figure S7: Functional analyses of the YnaI-MscS chimera.

a Electrophysiological experiments performed on MJF641 (Δ7) transformed with the YnaI-MscS chimera showed that showed a plateau is reached at ~50 pA, which might reflect the full opening of the chimera. The current depicted in the boxes represents the same cutout. On the right side, the all-point amplitude histogram corresponding to the shown trace is depicted with gaussian fitted curves shown. The gaussian fitting of the single channel openings (red curve) shows 50.5 ± 0.6 pA (maximum value) and 22 pA (FWHM, blue line). The green curve centered around 0 pA reflects the noise level. Only single channel openings were considered for the histogram. The bars are depicted with a binning of 2 pA. Experiments were conducted at +40 mV. **b** For the hypoosmotic downshock assay, cells were diluted into identical LB medium which had extra 0.3 M NaCl (control) or no additional NaCl (shock) and plated onto corresponding plates. After incubation, colonies were counted and the survival ratios determined. Triplicates were performed, and mean values and standard deviations were determined from the survival ratios of the triplicates (bottom), highlighting that the chimera provides protection of the cells during a hypoosmotic downshock. Source data are provided as a source data file.

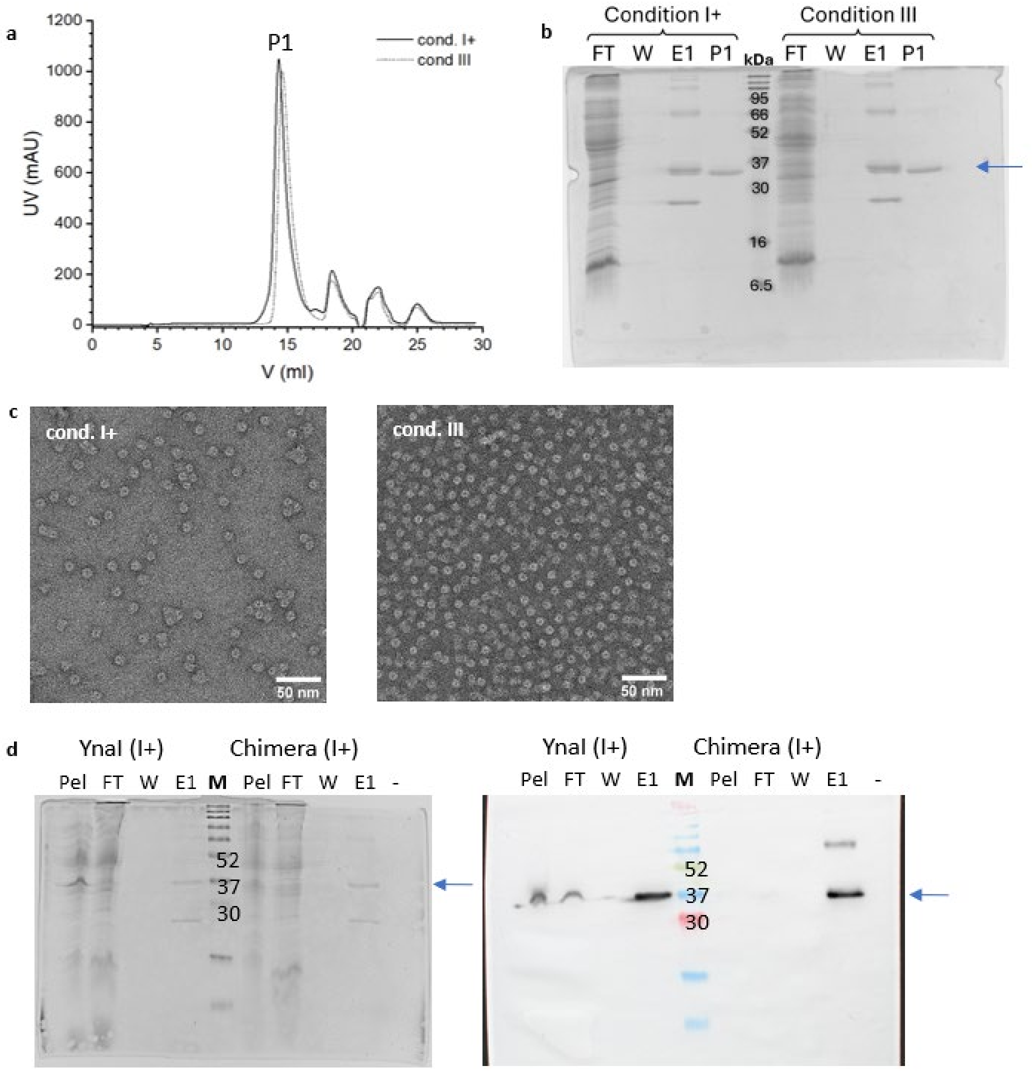

Figure S8: Purification of closed and open YnaI-MscS chimera.

The YnaI-MscS chimera was purified under conditions I+ and III. **a** The size exclusion chromatograms are shown for the chimera purified under condition I+ (standard DDM concentrations, solid line), and III (high DDM concentrations, dotted line), and the peak at 15 ml (P1) is the desired heptamer. **b** An SDS-PAGE is shown for the two purification approaches. Samples were taken of the IMAC flowthrough (FL), IMAC wash (W), IMAC protein-containing elution fraction (Elu), and HPLC heptamer peak (P1), as well as a Marker (kDa). The signals of the chimera at the height of the 37 kDa marker signal are highlighted with a blue arrow. **c** The P1 fractions of each purification were pooled and concentrated for subsequent cryo-EM preparation. Before, a dilution (1:3000) of the concentrated sample was negatively stained and imaged for quality assessment. **d** For comparison of the purifications of the chimera and of YnaI, two more SDS-PAGEs were run with identical application schemes and volumes. One was stained with Coomassie R-250 (left), and the other was used for Western blot analysis against the His_6 tag (right). A Penta-His HRP antibody was employed. The blue arrows highlight YnaI or the chimera, respectively. The Western blot shows signals of the His_6 tag in the cell pellet (Pel), flowthrough and wash fraction of YnaI but not the chimera, despite equal amounts of cells and samples. The highly concentrated IMAC elution fraction E1 shows a similarly strong signal for YnaI and the chimera (blue arrow). Conclusively, the chimera is less expressed (no signal from the cell pellet), but more efficiently purified than YnaI. Source data are provided as a source data file.

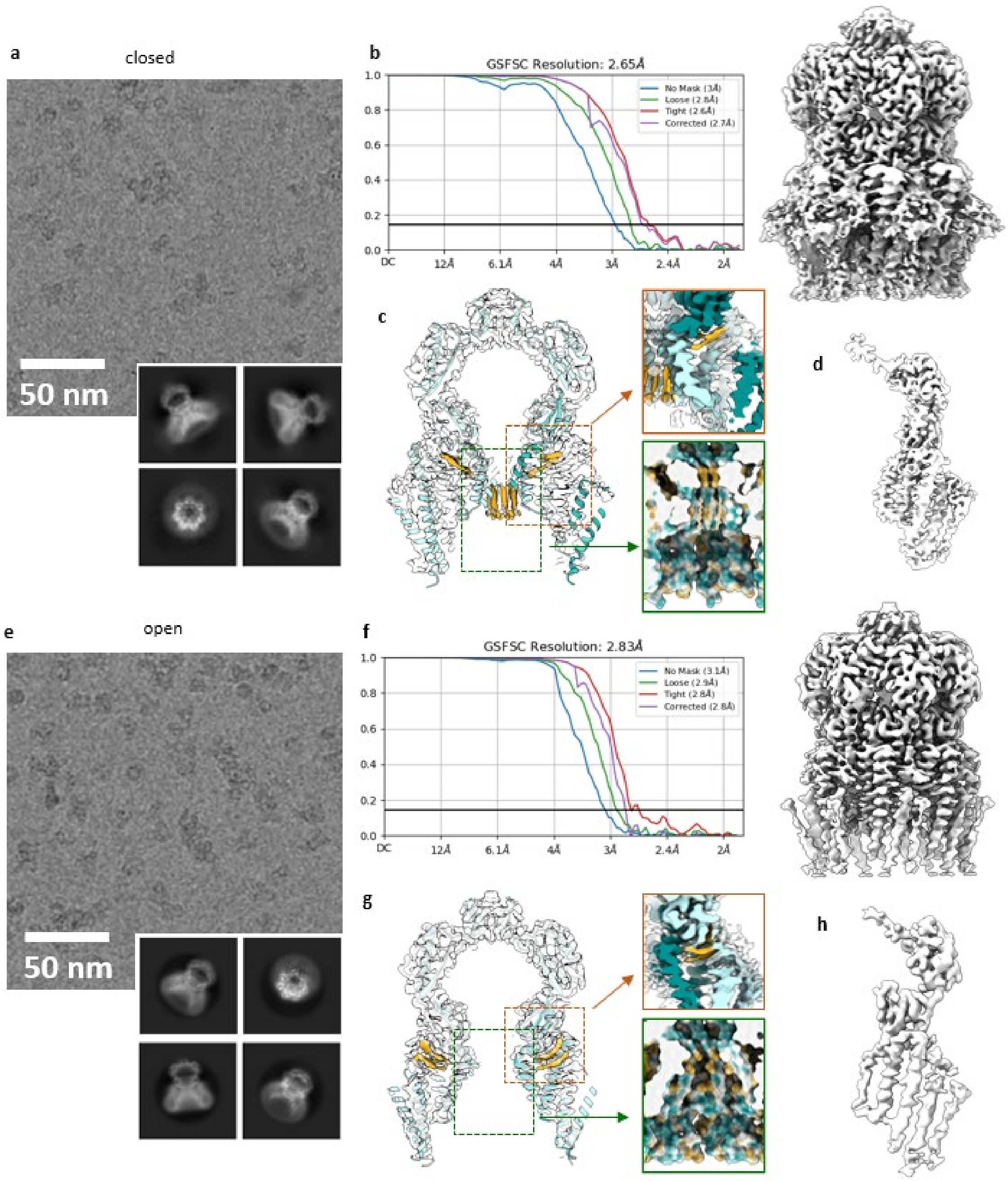

Figure S9: Cryo-EM analysis of the closed and open YnaI-MscS chimera.

The cryo-EM analysis is shown for both the closed and open chimera. **a** A representative micrograph of the data collection of the chimera purified under condition I+ is shown and four well represented 2D class averages. The edge of the box corresponds to 24.2 nm. **b** The final map had a resolution of 2.7 Å resolution. Data processing was done in cryoSPARC.[1] **c** In a central slice, the model is coloured in light blue with one subunit darker, and lipid densities are depicted in orange. The inserts in the boxes are shown enlarged at the right side for the hydrophobic pockets (orange box) and the pore lipids (green box). The latter are shown with the model surface couloured according to its hydrophobicity. Pore lipids are highlighted with a white silhouette. **d** To improve resolution and map quality of helix TM(-2), data was imported into Relion 5[3] and a refinement with C7 symmetry relaxation and blush denoising was conducted. The resulting density of the best resolved subunit is shown. **e** A representative micrograph of the data collection of the chimera purified under the condition III is shown and four well represented 2D

class averages. The edge of the box corresponds to 24.2 nm. **f** The final map had a resolution of 2.8 Å resolution, but the transmembrane part is notably worse resolved compared to the closed chimera. **g** In a central slice, the model is coloured in light blue with one subunit darker, and lipid densities are depicted in orange. The inserts in the boxes are shown enlarged at the right side. The hydrophobic pockets are shown (orange box). No pore lipids are observed (green box), the model surface is coloured according to its hydrophobicity. **h** To improve resolution and map quality of helix TM(-2), data was imported into Relion 5 and a refinement with C7 symmetry relaxation and blush denoising was conducted. The resulting auto-sharpened density of the best resolved subunit is shown. The helical backbones of all helices are clearly visible, yet the density of the outermost two helices is not good enough to identify side chains.

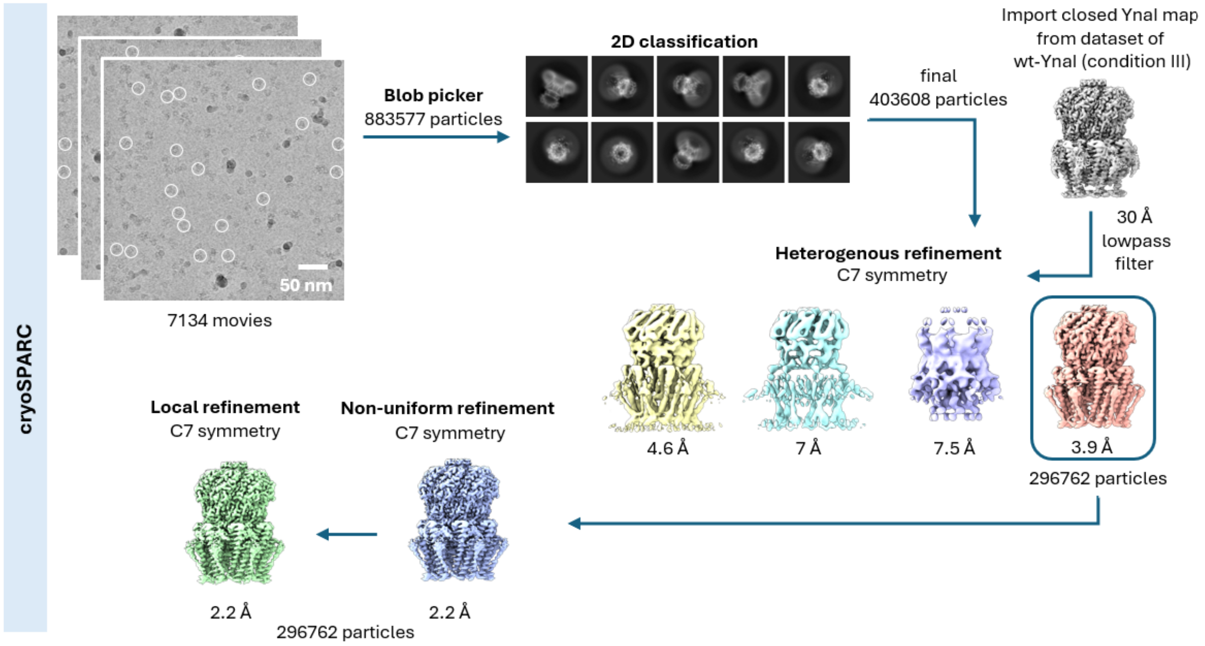

Figure S10: Cryo-EM workflow of closed YnaI.

The cryo-EM image processing workflow is shown for closed YnaI, which was obtained upon purification under condition I+ (standard DDM concentrations and additional lipids). It resulted in a final map with 2.2 Å resolution. Processing was done in cryoSPARC.[1]

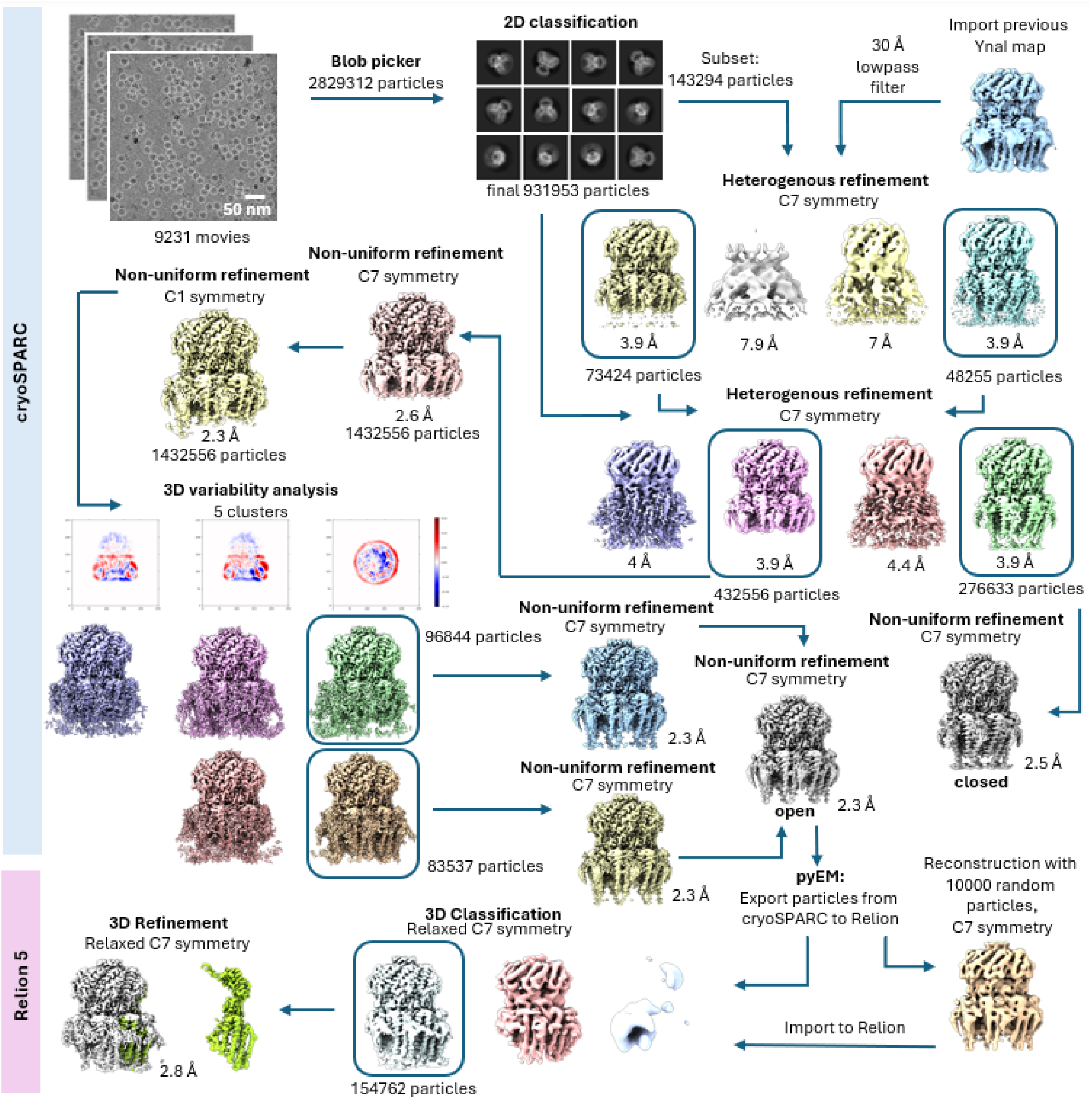

Figure S11: Cryo-EM workflow of open YnaI.

The cryo-EM image processing workflow is shown for YnaI, which was obtained upon purification under condition III (higher DDM concentrations). It resulted in a final closed map with 2.5 Å resolution and an open map with 2.3 Å resolution in cryoSPARC[1]. The open YnaI was further processed in Relion[3] with relaxed C7 symmetry, resulting in a map with 2.8 Å resolution that resolves one subunit better than the map obtained with C7 symmetry.

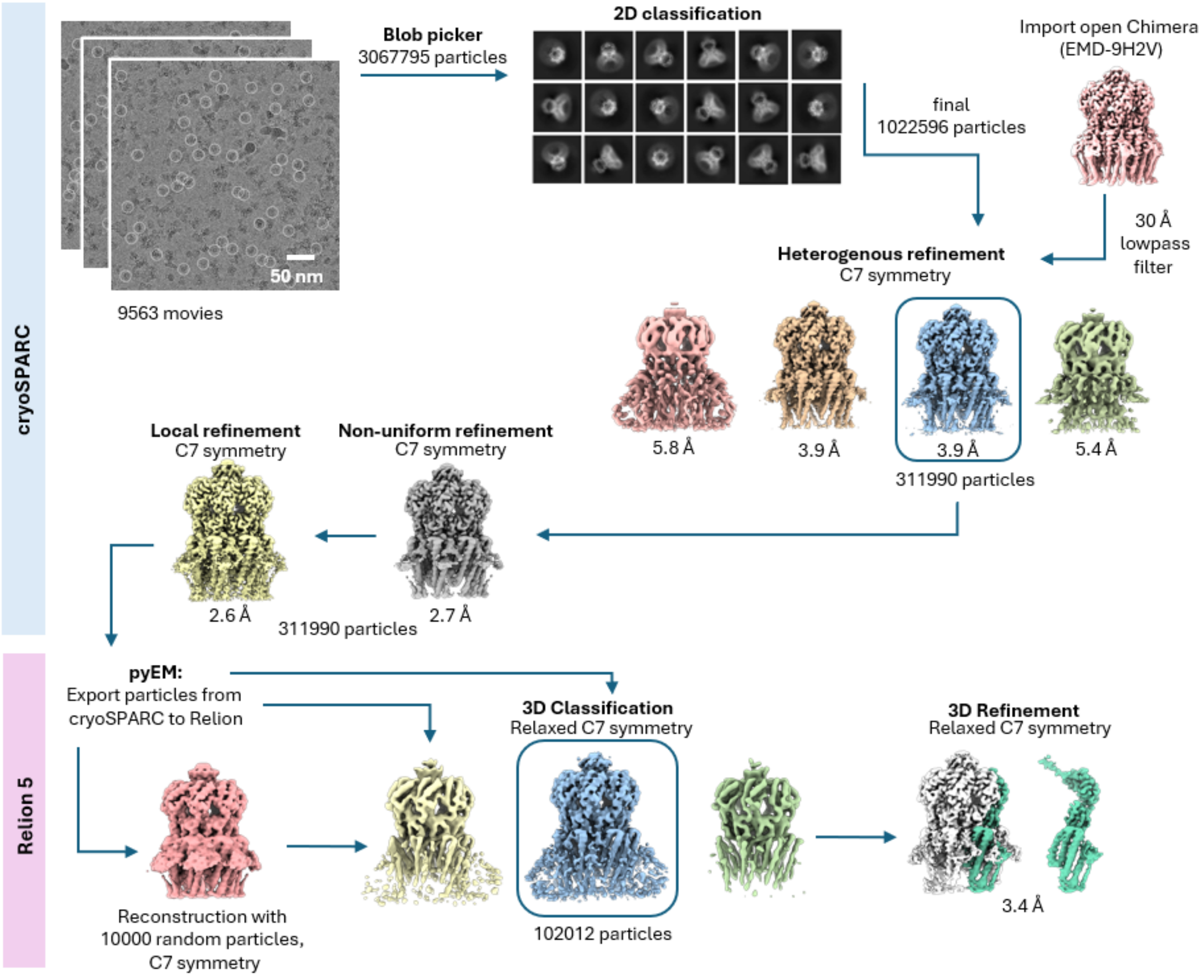

Figure S12: Cryo-EM workflow of the closed YnaI-MscS chimera.

The cryo-EM image processing workflow is shown for the YnaI-MscS chimera, which was obtained upon purification under condition I+ (standard DDM concentrations and additional lipids). It resulted in a final map with 2.6 Å resolution in cryoSPARC[1]. The map was further processed in Relion[3] with relaxed C7 symmetry, resulting in a map with 3.4 Å resolution that resolves one subunit better than the map obtained with C7 symmetry.

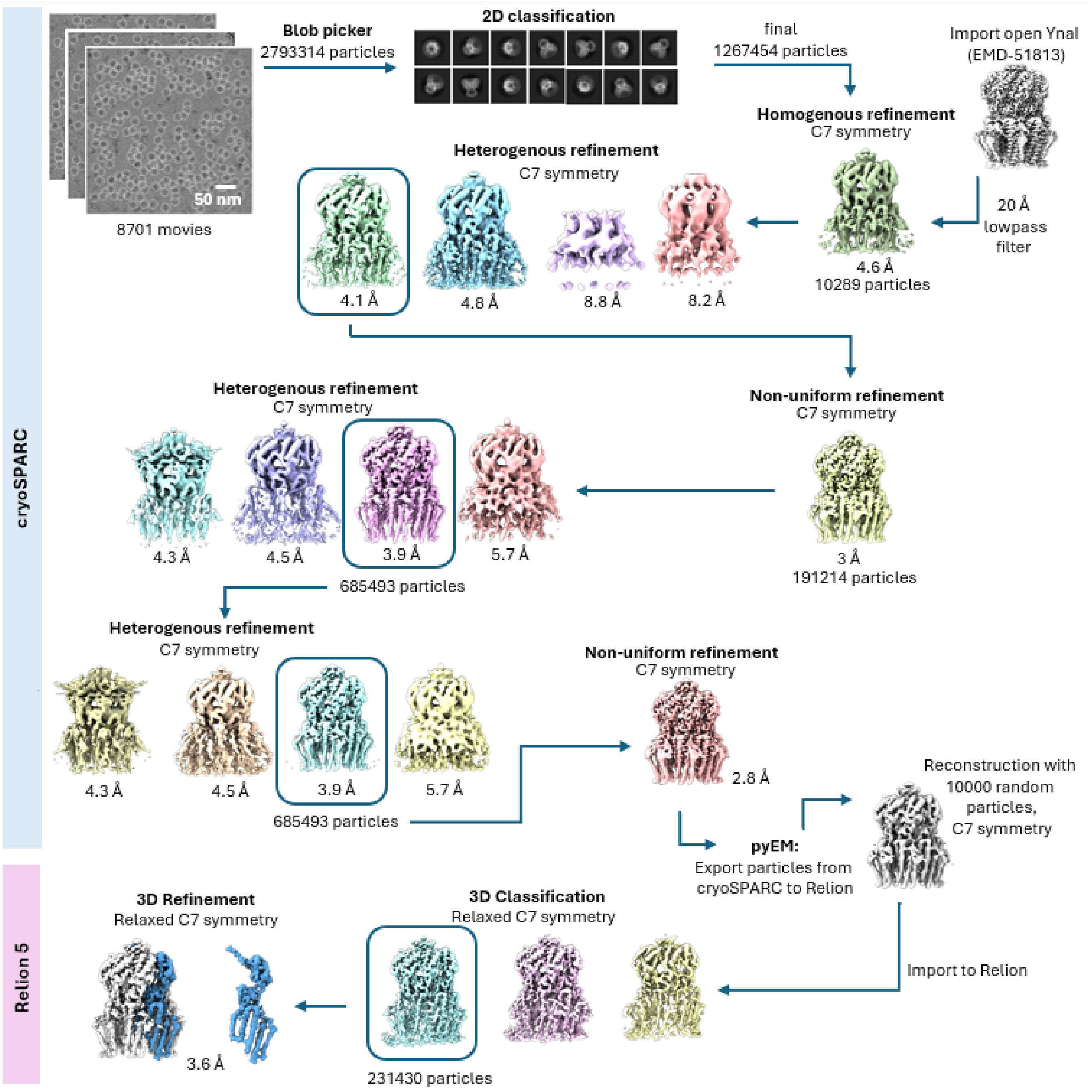

Figure S13: Cryo-EM workflow of the open YnaI-MscS chimera.

The cryo-EM image processing workflow is shown for the YnaI-MscS chimera, which was obtained upon purification under condition III (higher DDM concentrations). It resulted in a final map with 2.8 Å resolution in cryoSPARC[1]. The map was further processed in Relion[3] with relaxed C7 symmetry, resulting in a map with 3.6 Å resolution that resolves one subunit better than the map obtained with C7 symmetry.

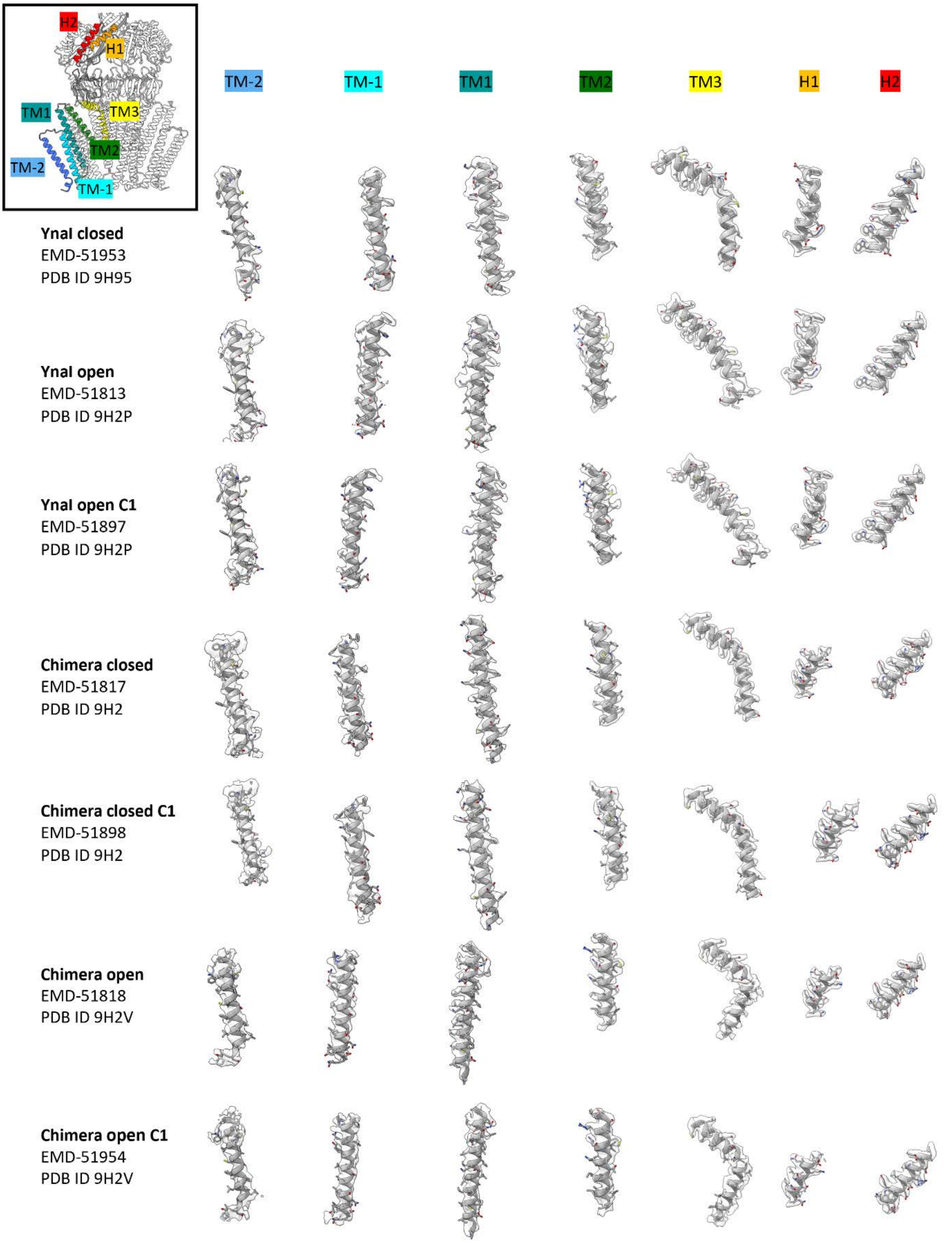

Figure S14: Presentations of maps and models of the helices.

The figure in the upper left depicts closed YnaI with the helices highlighted and labelled. The models of the helices are shown in the respective maps. The sample name, and EMDB- and PDB accession codes are given on the left.

Table 1a: Cryo-EM data collection, refinement and validation statistics for closed and open WT-YnaI

	YnaI closed<br>**EMD-51953**<br>**PDB ID 9H95**	YnaI open<br>**EMD-51813**<br>**PDB ID 9H2P**	YnaI open<br>sym-exp.<br>**EMD-51897**
**Data collection and processing**			
Magnification	75 kx	75 kx	
Voltage (kV)	300 kx	300 kx	
Electron exposure (e–/Å^2)	40	40	
Defocus range (μm)	-0.6 to -1.4	-1.0 to -1.8	
Pixel size (Å)	0.946	0.946	
Symmetry imposed	C7 (cryoSPARC)	C7 (cryoSPARC)	C7-relaxed (Relion 5)
Initial particle images (no.)	883577	2829312	180381
Final particle images (no.)	296762	180381	151762
Map resolution (Å)	2.2	2.3	2.8
FSC threshold	0.143	0.143	0.143
Map resolution range (Å)	2-4	2-4	2-4
**Refinement**			
Initial model used (PDB code)	6URT	9H95	
Model resolution (Å)	2.4	2.1	
FSC threshold	0.143	0.143	
Map sharpening *B* factor (Å^2)	-10	-30	
Model composition			
Non-hydrogen atoms	19201	20076	
Protein residues	2324	2324	
Ligands	56	42	
B factors (Å^2)			
Protein	38	38	
Ligand	85	83	
R.m.s. deviations			
Bond lengths (Å)	0.003	0.002	
Bond angles (°)	0.561	0.378	
Validation			
MolProbity score	1.5	1.95	
Clashscore	5.86	20.96	
Poor rotamers (%)	1.74	0.35	
Ramachandran plot			
Favored (%)	98.79	97.27	
Allowed (%)	1.21	2.73	
Disallowed (%)	0.00	0.00	

Table 1b: Cryo-EM data collection, refinement and validation statistics for closed and open YnaI-MscS chimera

	Chimera closed **EMD-51817** **PDB ID 9H2S**	Chimera closed sym-exp **EMD-51898**	Chimera open **EMD-51818** **PDB ID 9H2V**	Chimera open sym-exp. **EMD-51954**
**Data collection and processing**				
Magnification	75 kx		75 kx	
Voltage (kV)	300 kx		300 kx	
Electron exposure (e–/Å^2)	70		70	
Defocus range (μm)	-0.6 to -1.4		-0.6 to -1.6	
Pixel size (Å)	0.946		0.946	
Symmetry imposed	C7 (cryoSPARC)	C7-relaxed (Relion 5)	C7 (cryoSPARC)	C7-relaxed (Relion 5)
Initial particle images (no.)	3067795	311990	2793314	283615
Final particle images (no.)	311990	102012	283615	231430
Map resolution (Å)	2.7	3.4	2.8	3.6
FSC threshold	0.143	0.143	0.143	0.143
Map resolution range (Å)	2-4	2-5	2-4	2-5
**Refinement**				
Initial model used (PDB code)	9H95 and 6RLD		9H2S	
Model resolution (Å)	2.9		3.0	
FSC threshold	0.143		0.143	
Map sharpening *B* factor (Å^2)	-30		-30	
Model composition				
Non-hydrogen atoms	18235		18032	
Protein residues	2289		2268	
Ligands	21		21	
B factors (Å^2)				
Protein	76		56	
Ligand	146		153	
R.m.s. deviations				
Bond lengths (Å)	0.002		0.002	
Bond angles (°)	0.397		0.376	
Validation				
MolProbity score	1.98		2.28	
Clashscore	25.94		35.18	
Poor rotamers (%)	0.87		1.09	
Ramachandran plot				
Favored (%)	97.63		96.58	
Allowed (%)	2.37		3.42	
Disallowed (%)	0.00		0.00	

References

1. Punjani, A., Rubinstein, J. L., Fleet, D. J. & Brubaker, M. A. cryoSPARC: algorithms for rapid unsupervised cryo-EM structure determination. *Nat. Methods* **14**, 290–296 (2017).

2. Forsberg, B. O., Shah, P. N. M. & Burt, A. A robust normalized local filter to estimate compositional heterogeneity directly from cryo-EM maps. *Nat. Commun.* **14**, 5802 (2023).

3. Scheres, S. H. W. RELION: Implementation of a Bayesian approach to cryo-EM structure determination. *J. Struct. Biol.* **180**, 519–530 (2012).

4. Pettersen, E. F. *et al.* UCSF ChimeraX: Structure visualization for researchers, educators, and developers. *Protein Sci.* **30**, 70–82 (2021).

5. Goddard, T. D. *et al.* UCSF ChimeraX: Meeting modern challenges in visualization and analysis. *Protein Sci.* **27**, 14–25 (2018).
