# Supplementary figures and images for: Mechanosensitive channel engineering: A study on the mixing and matching of YnaI and MscS sensor paddles and pores
Source: Nat Commun. 2025 Aug 23;16:7881. doi: 10.1038/s41467-025-63253-0 (PMC12375004; doi:10.1038/s41467-025-63253-0)

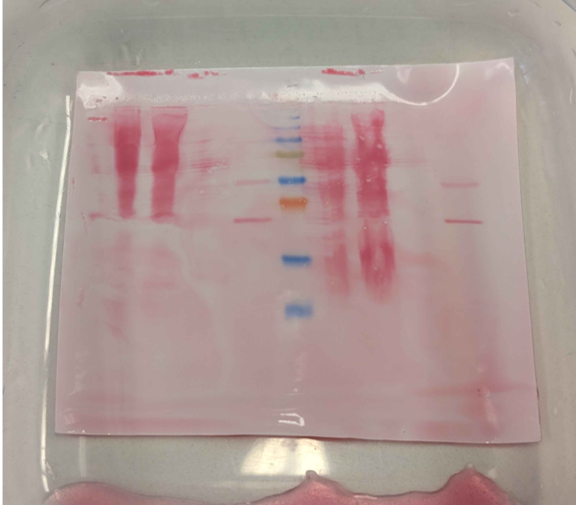

Supplement: Supplementary file 4 — Source Data [file 41467_2025_63253_MOESM4_ESM.zip › S8d_ponceau-s.png]

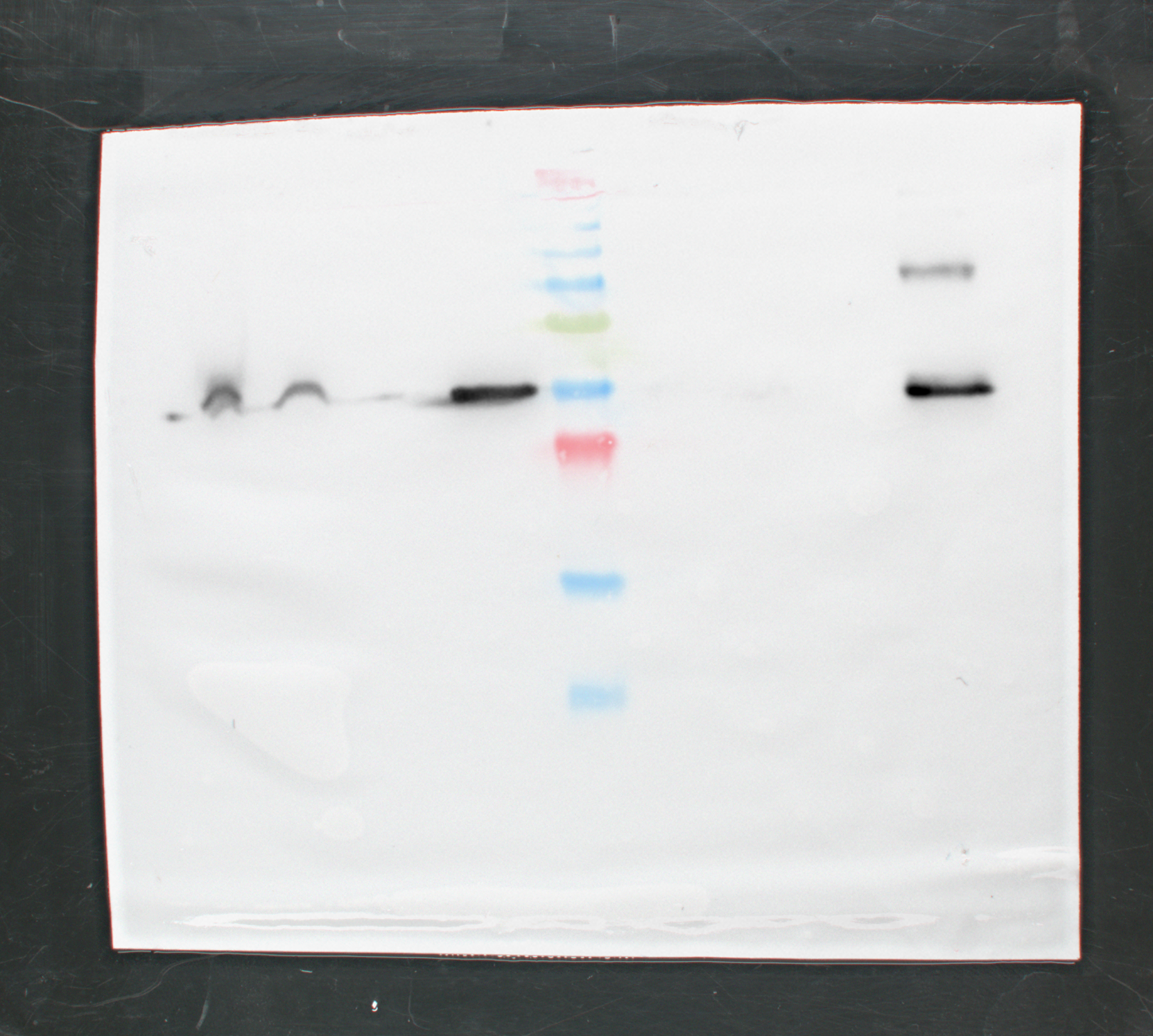

Supplement: Supplementary file 4 — Source Data [file 41467_2025_63253_MOESM4_ESM.zip › S8d_WesternBlot.png]

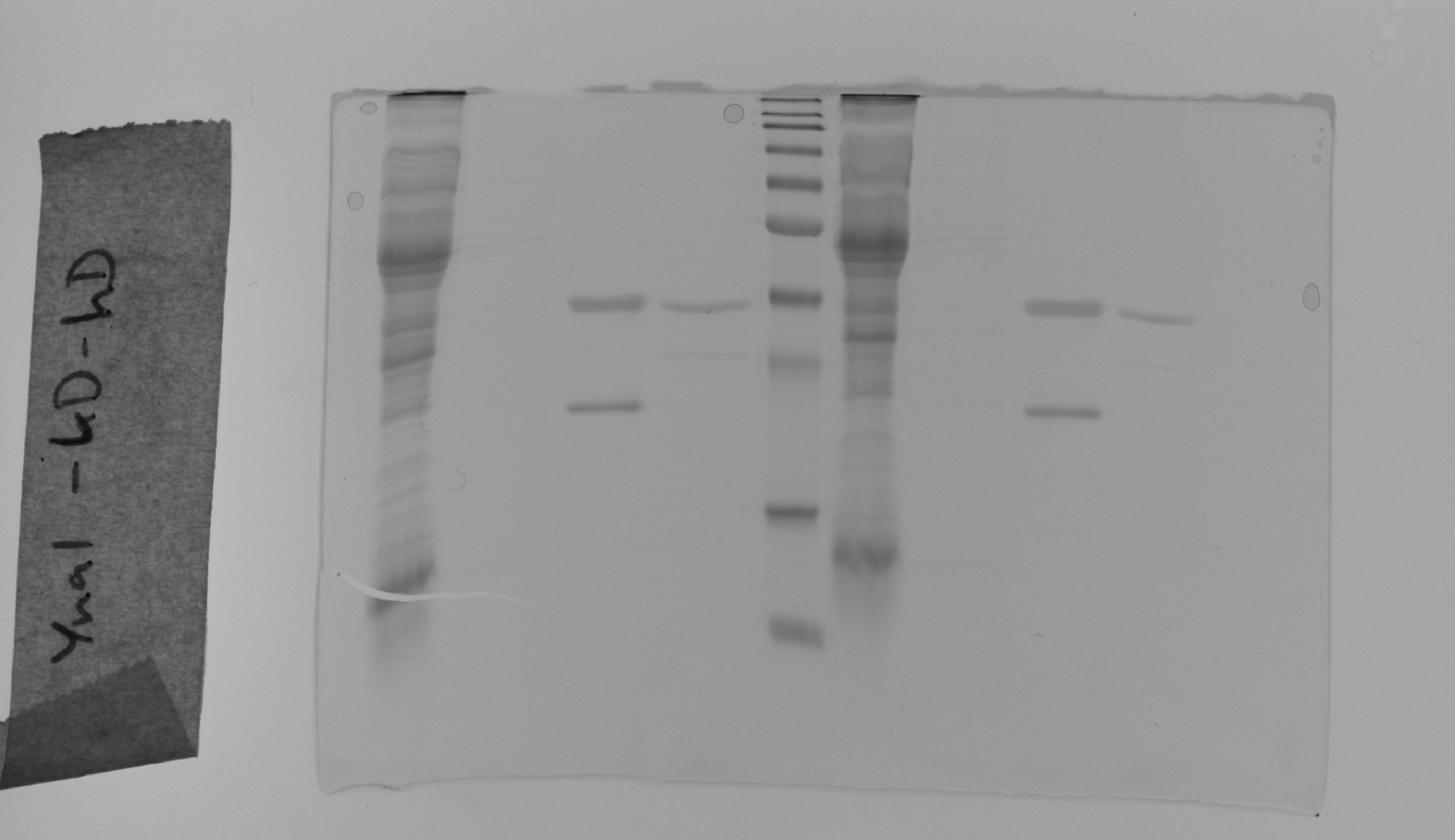

Supplement: Supplementary file 4 — Source Data [file 41467_2025_63253_MOESM4_ESM.zip › S2b.png]

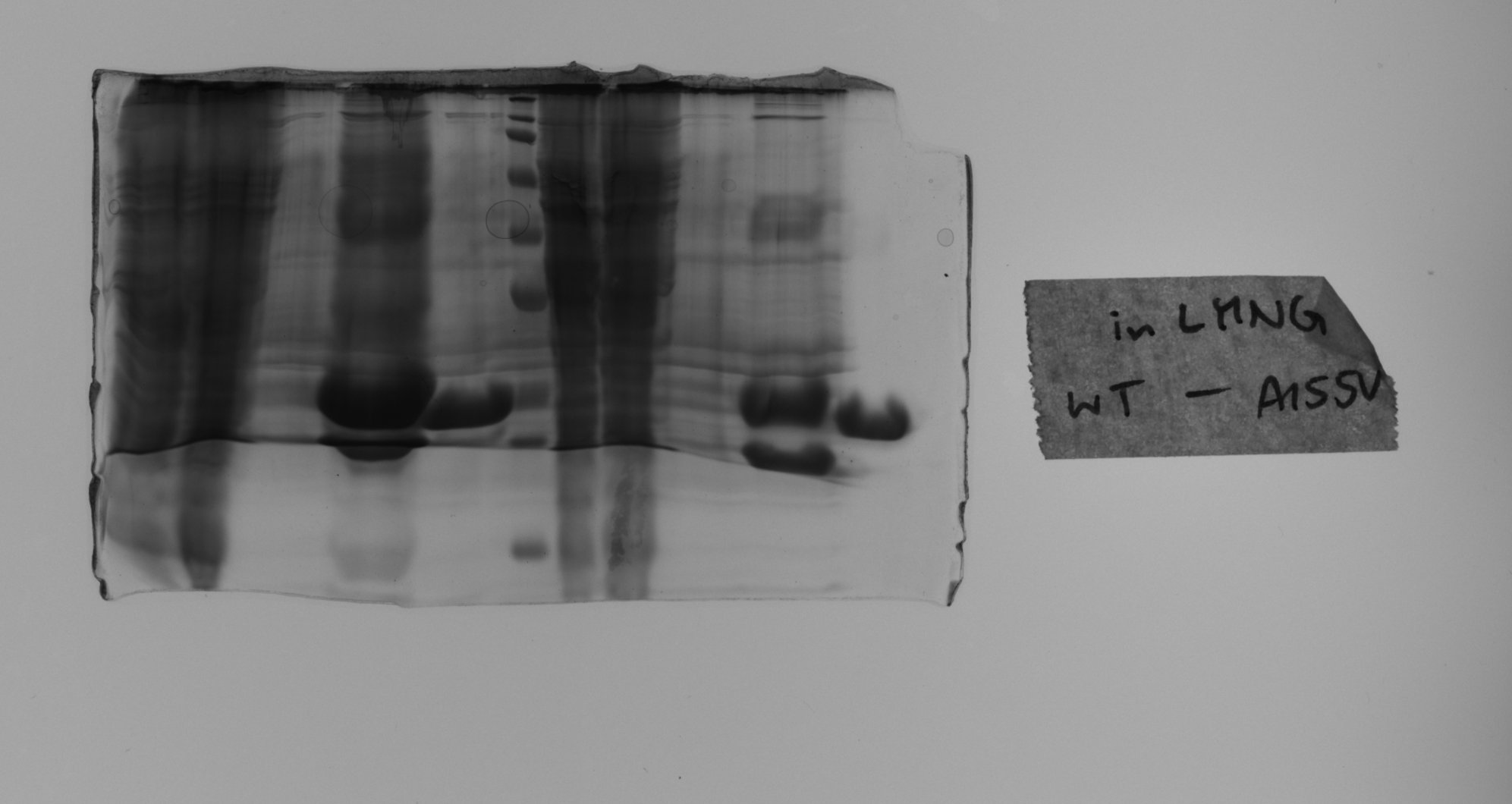

Supplement: Supplementary file 4 — Source Data [file 41467_2025_63253_MOESM4_ESM.zip › S2b-2.png]

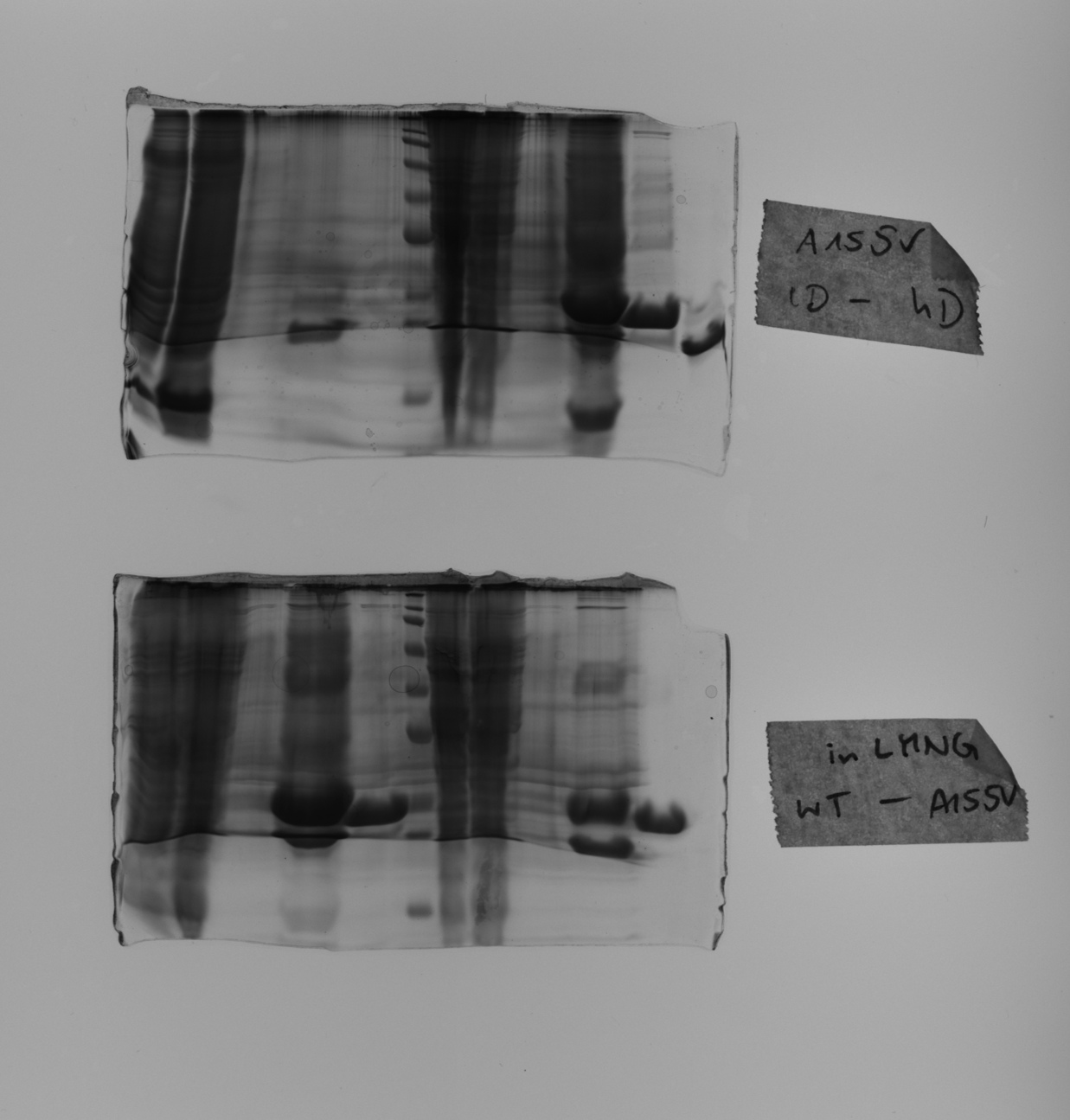

Supplement: Supplementary file 4 — Source Data [file 41467_2025_63253_MOESM4_ESM.zip › S4b.png]

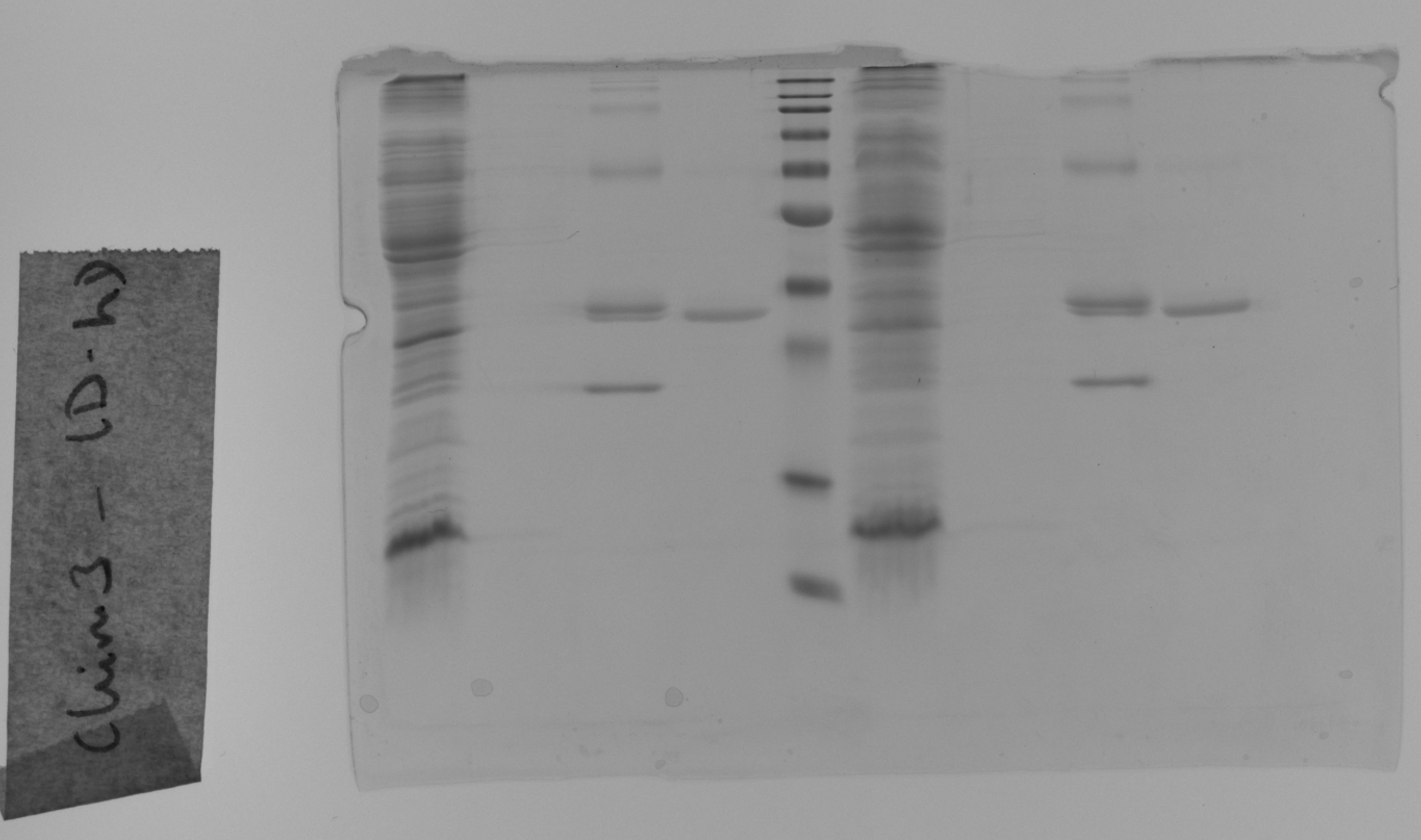

Supplement: Supplementary file 4 — Source Data [file 41467_2025_63253_MOESM4_ESM.zip › S8b.png]

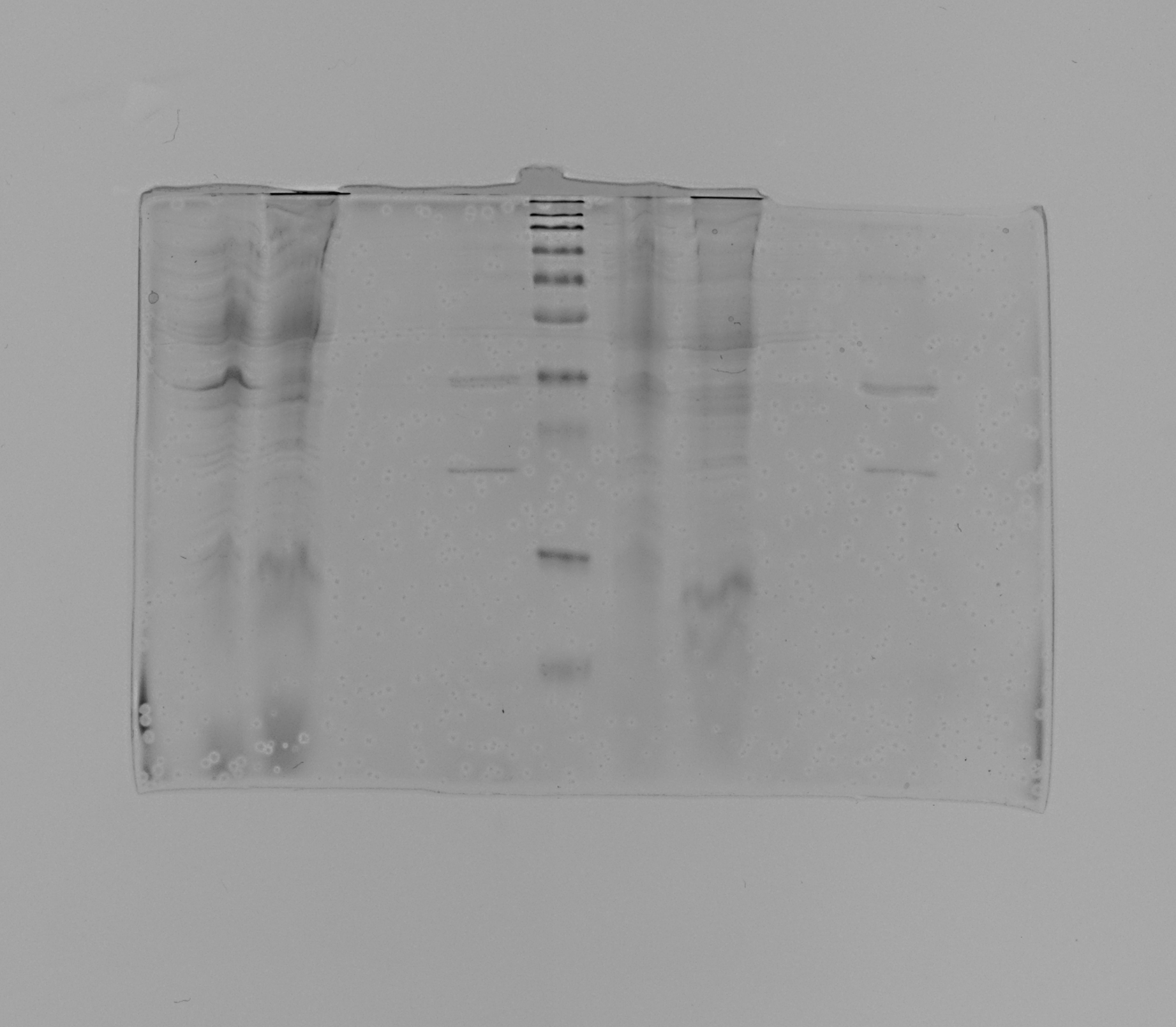

Supplement: Supplementary file 4 — Source Data [file 41467_2025_63253_MOESM4_ESM.zip › S8d_coomassie.png]
